# The value of magnetic resonance spectroscopy as a supplement to MRI of the brain in a clinical setting

**DOI:** 10.1371/journal.pone.0207336

**Published:** 2018-11-15

**Authors:** Jussi Hellström, Romina Romanos Zapata, Sylwia Libard, Johan Wikström, Francisco Ortiz-Nieto, Irina Alafuzoff, Raili Raininko

**Affiliations:** 1 Department of Radiology, Uppsala University, Uppsala, Sweden; 2 Department of Immunology, Genetics and Pathology, Uppsala University, Uppsala, Sweden; 3 Department of Pathology, Uppsala University Hospital, Uppsala, Sweden; McLean Hospital, UNITED STATES

## Abstract

**Background:**

There are different opinions of the clinical value of MRS of the brain. In selected materials MRS has demonstrated good results for characterisation of both neoplastic and non-neoplastic lesions. The aim of this study was to evaluate the supplemental value of MR spectroscopy (MRS) in a clinical setting.

**Material and methods:**

MRI and MRS were re-evaluated in 208 cases with a clinically indicated MRS (cases with uncertain or insufficient information on MRI) and a confirmed diagnosis. Both single voxel spectroscopy (SVS) and chemical shift imaging (CSI) were performed in 105 cases, only SVS or CSI in 54 and 49 cases, respectively. Diagnoses were grouped into categories: non-neoplastic disease, low-grade tumour, and high-grade tumour. The clinical value of MRS was considered very beneficial if it provided the correct category or location when MRI did not, beneficial if it ruled out suspected diseases or was more specific than MRI, inconsequential if it provided the same level of information, or misleading if it provided less or incorrect information.

**Results:**

There were 70 non-neoplastic lesions, 43 low-grade tumours, and 95 high-grade tumours. For MRI, the category was correct in 130 cases (62%), indeterminate in 39 cases (19%), and incorrect in 39 cases (19%). Supplemented with MRS, 134 cases (64%) were correct, 23 cases (11%) indeterminate, and 51 (25%) incorrect. Additional information from MRS was beneficial or very beneficial in 31 cases (15%) and misleading in 36 cases (17%).

**Conclusion:**

In most cases MRS did not add to the diagnostic value of MRI. In selected cases, MRS may be a valuable supplement to MRI.

## Introduction

In clinical practice, magnetic resonance spectroscopy (MRS) has most often been used as a complementary method in cases in which other methods have not given sufficient information for diagnosis and adequate treatment. In such cases, interpretation of MRS findings is much more difficult than in most scientific projects, in which MRS is used in selected cases with limited numbers of alternative diagnoses to answer well-defined questions.

MRS has most often been used in tumour diagnostics. It has been shown to improve the differential diagnosis of brain tumours in conjunction with magnetic resonance imaging (MRI) [1–5]. However, these studies have often been focused on a specific question, e.g. high-grade versus low-grade glioma, in materials only consisting of neoplasms, even only of gliomas. Other reports have suggested that MRS does not provide sufficient information to grade gliomas accurately [6–9]. MRS has, however, been shown to differentiate between neoplastic and non-neoplastic lesions with a high sensitivity and specificity [10,11] and a higher accuracy than MRI (78% versus 66%) [12]. There are no large MRS studies in a clinical setting in which the type of lesion is unknown and the number of alternative diagnoses is not limited. Since both acquisition and analysis of MRS data are time-consuming, it is crucial that the clinical value is established.

The aim of this study was to evaluate how much additional information MRS gives in comparison to MRI in non-selected clinical patients.

## Materials and methods

### Materials

The study plan was approved by the Uppsala regional ethics committee. The clinical archive of the radiology department (i.e. examinations for research purposes were not included) was searched for MR spectroscopy of the brain performed from January 2004 to April 2014, yielding 443 examinations. To use patient data from the hospital archive for research purposes, written informed consent was needed from all living patients and parents of underage patients. The medical records were checked for a definitive diagnosis, valid at the time of the MRS. Cases with uncertain diagnoses, including patients who underwent follow-up at other hospitals, and cases in which spectroscopic analysis or raw data were not available were excluded. Cases with a low spectral quality were excluded from the study.

This left a total of 208 examinations to be included in the study. The material consisted of 186 patients: 103 men and 83 women, age range 0–84 years (median 43 years). Fifteen patients had undergone repeated examinations because of a new lesion or altered appearance of a known lesion. The clinical indications for the examinations are shown in Table 1.

**Table 1:** Indications for MRS.

Clinical question	No. of cases
Recurrent tumour versus reaction to irradiation/chemotherapy	69
Tumour grading	56
Neoplastic vs non-neoplastic lesion (infection, inflammation, ischaemia, etc.)	29
Lesion of unknown aetiology on MRI	23
Metastatic disease versus primary tumour	11
Type of a non-neoplastic lesion	6
Metabolic disease	5
Epileptic focus	4
Pre-radiotherapy (Extent of the pathologic area)	4
Biopsy planning (Hot spot)	1
**Total**	**208**

### Radiological evaluation

An experienced neuroradiologist (licensed neuroradiologist for 35 years, experience with MRI 32 years and with MRS 20 years), blinded to the definitive diagnoses, reviewed both the MR images and the MRS curves and other MRS analyses. Evaluation of the examinations was performed in the same way as is done in the clinical routine at our hospital. All radiological examinations obtained before and at the time of MRS were available. Clinical information to the radiologist by the referring clinician was used if available. MR images were evaluated first without and then with MRS data.

### Radiological technique

The MR imaging technique varied over the years and according to the indication for the examination. Diffusion-weighted images were often included, and perfusion MRI has been performed routinely in cases of suspected tumour for the last 5 years. Proton MRS technique was individualized according to MRS indication and case specific questions. The patient´s clinical state, co-operation and total MR examination time were also taken in account. We tried to avoid anaesthesia. The standard technique was a combination of SVS using a long TR and a short TE and CSI using a semi-long TE. SVS using a short TE was chosen to better demonstrate metabolites like myoinositol and lipids. CSI was used to analyse a larger area and to demonstrate the extent and heterogeneity of the pathologic area, e.g. locations of hot spots before biopsy. Both single voxel spectroscopy (SVS) and chemical shift imaging (CSI) were successively performed in 105/208 examinations. Only SVS was performed in 54 examinations and only CSI in 49 examinations. In 2004–2005, a Philips Intera (Philips Healthcare, Best, the Netherlands) imager was mainly used. Since 2005, the spectra were obtained with a Siemens Avanto (Siemens Medical Systems, Erlangen, Germany) imager. Both imagers operated at 1.5 T. In a single examination in 2009, a Philips Intera 3 T system was used.

At SVS, the voxel was placed in the lesion seen on MRI or in a clinically suspected area. The size and form of the voxel was individually adjusted in order to select a representative sample. The average voxel volume was 5.0 cm^3^. The smallest voxel in this material was 0.72 cm^3^ but voxel volumes less than 1 cm^3^ were only exceptionally used. If there was contrast enhancement, the voxel was placed in that area. The voxel was placed in solid tissue, avoiding necrotic areas and cerebrospinal fluid. In suspected metabolic diseases without MRI changes, the voxel was placed in the white matter, grey matter, or both depending on the suspected disease. If MRI did not reveal pathological areas one voxel was routinely placed in supraventricular white matter and the other voxel in deep grey matter (basal ganglia/thalamus). If the clinician suspected a certain disease or disease group one voxel was placed in the region which most likely could be abnormal in such diseases and the other in a less suspect region. A control voxel in the normal or suspected normal area was not used in all cases and never if CSI was also performed. A control material of healthy volunteers with the same SVS technique and analysis method was utilized in evaluations.

Fat contamination and areas with susceptibility disturbances were avoided. To detect the areas with susceptibility disturbances, T2*-weighted gradient echo or SWI sequences were used before MRS in patients who had undergone operations or had a lesion close to the skull base.

For SVS, point resolved spectroscopy (PRESS) sequences were used. The repetition time/ echo time (TR/TE) was 6000/20–22 ms (Philips Intera 1.5T), 5000/30 ms (Siemens Avanto) or 5000/35 ms (Philips Intera 3T). In 10 cases, modifications of the technical settings were made, often with a shortening of the TR or using a semi-long TE as a complement to the routine sequence. Sixteen unsuppressed water reference acquisitions were obtained for quantifications. An unsuppressed water signal was used as an internal reference when metabolite concentrations were estimated. The data were processed using the LCModel. Routinely, we restricted the model to the range 0.2–4.0 ppm. The spectra were corrected for eddy currents. All spectra were manually assessed to exclude obvious non-randomness in the residuals or erroneous assignment of metabolites. Post-processing was made by an MR physicist. Examinations of low spectral quality, e.g. those with signal-to-noise ratio (SNR) <5, were not included in the material. The metabolites included in the diagnostic analyses had a Cramer-Rao lower bound (CRLB) ≤20 with exception of lactate. The presence of lactate was considered to be real if there was a clear inverted doublet peak in the spectrum with a semilong TE even if the CRLB was >20. Millimolar metabolite concentrations (mM, millimoles/liter substance) were measured using tissue water as a reference. Ratios were routinely calculated using total creatine (Cr) as a reference but also other ratios, like N-acetylaspartate/choline (NAA/Cho), were used as diagnostic tools. We mainly used metabolite ratios since the absolute concentrations are calculated assuming that water concentration of the brain tissue is constant, which cannot always be expected.

On CSI, the examined area covered the pathological area but also normal or suspected normal contralateral tissue. On CSI, the TE was chosen so that the lactate peak pointed downwards. A TR of 1500–2500 ms and a TE of 135–144 ms were used. The section thickness was 15 mm and the nominal voxel sizes 10x10x15 mm^3^. Saturation bands were placed for suppression of osseous, fatty and air-containing structures in surroundings. All data post-processing was performed by an MR physicist with softwares provided by the MR imager manufacturers and spectra at 1.1–3.5 ppm were analysed. All voxels were analysed but those with low spectral quality were not used for diagnostic purposes. In good quality spectra, at least the upper halves of the choline peaks could be separated from the creatine peaks. Metabolite ratios to Cr and Cho were calculated and used in diagnostic analyses. Colour maps overlaid on the anatomical images were made routinely for metabolites Cho, Cr, NAA and lactate and for metabolite ratios using creatine and choline as references.

MRS interpretations were made in accordance with the criteria in literature. For differentiation between low-grade (I-II) and high-grade (III-IV) tumours, a Cho/NAA threshold of 1.66 and Cho/Cr threshold of 1.56 has been suggested on CSI [6]. Presence of lipids and lactate and a reduced NAA peak have been found to indicate a grade IV tumour [13]. For the differentiation between tumour recurrence and radiation reaction, a Cho/Cr ratio > 1.5 [14], 1.8 [15] to 2 [16] has been shown to predict tumour on CSI, while a Cho/Cr threshold of 1.1 [17] has been suggested on SVS. The principles of the MRS analysis are summarised in supporting information S1 Table.

In the cases in which the MRS analysis results were lost but raw data were saved, a new analysis was performed.

### Confirmation of definitive diagnoses

The diagnoses on MRI and MRS were compared with the definitive diagnosis. The methods used for confirmation are shown in Table 2.

**Table 2:** Confirmation of the definitive diagnosis.

Method	No. of cases
Neuropathological	104
Long-term follow-up^a^	91
Laboratory test including genetic testing	9
Intracranial/extracranial EEG^b^	2/2
**Total**	**208**

^a^The long-term follow-up was at least 6 months.

^b^Patients having epilepsy

The tissue samples for neuropathological analyses were obtained by stereotactic needle biopsy (45 cases), by resection during open surgery (58 cases), or at autopsy (1 case). The median time from radiological examination to sampling for neuropathological analyses was 21 days (range 0–280 days, upper quartile 56 days). The possible effect of the time elapsed from the radiological examinations to sampling was considered for each case. Longer time intervals were accepted in benign and slowly altering diseases. The material was processed at the department of pathology following a standard routine including the use of histochemical (haematoxylin-eosin (HE)) and immunohistochemical techniques. All original HE-stained slides were reassessed by two neuropathologists. The grading of tumours followed the World Health Organization (WHO) Classification of Tumours of the Central Nervous System [18]. Briefly, all cases with at least mitotic figures in HE sections were graded as high-grade tumours. The diagnosis was also considered as a histologically verified recurrent tumour in the 18 patients with a histologically confirmed high-grade glioma at primary operation, followed by a fast-growing lesion in the same location and a rapid deterioration of clinical condition.

In some patients, a diagnosis was made during a follow-up of at least 6 months, during which time imaging findings, other examinations, and clinical condition were evaluated. In this study, the final diagnosis”reaction to irradiation and/or chemotherapy” was based on a long-term follow-up, of at least 6 months, with stable or vanishing lesion, together with favourable clinical outcome.

### Classification of findings

For statistical comparisons, the diagnoses were divided into three clinically relevant categories: primary high-grade CNS tumours (WHO grades III–IV) or metastasis, low-grade tumours (WHO grades I–II), and non-neoplastic lesions/diseases. The MRI and MRS diagnoses were classified as follows:

- Correct category
- Indeterminate: The diagnosis was categorised correctly, but also an incorrect category was given as an alternative.
- Incorrect category

Additional comparisons were made for some subcategories or exact diagnoses.

To evaluate the clinical benefits of MRS, the reviewers’ reports for MRI with and without MRS supplementation were compared to determine which report was most helpful to the referring clinician. The additional information from the spectroscopic report was labelled as follows:

- Very beneficial: The MRS report gave a correct diagnosis/diagnostic category or correct spatial location for a lesion, whereas the MRI report did not.
- Beneficial: The MRS report ruled out one or some of the earlier suspected diseases/conditions or was more specific than the MRI report.
- Inconsequential: Same level of diagnostic information in both reports.
- Misleading: The spectroscopic report contained less or incorrect information.

The results using the different methods and diagnostic categories were compared using a **χ**^**2**^ test.

### Interobserver agreement

Another experienced neuroradiologist (licensed neuroradiologist for 13 years, experience with MRI 19 years and with MRS 10 years), blinded to the reports of the first reader, reviewed MRI and MRS in the same way as the first reader in 50 randomly chosen cases. The interpretations of the two readers were compared to determine whether the core statements of the reports were in agreement. The interpretations were considered to be in agreement if the diagnoses fell into the same diagnostic category or both readers had given an indeterminate interpretation.

## Results

For the interobserver agreement for MRI, there was an agreement in 37 cases (74%) and for MRS in 38 cases (76%). κ-coefficients were 0.64–0.65, representing good agreement.

One-half of the definitive diagnoses were neuropathologically verified (Table 2). The distribution of the diagnoses into categories is shown in Table 3. About two-thirds of the lesions were neoplastic, and about two-thirds of the tumours were high-grade. The most common group in non-neoplastic diagnoses was reaction to irradiation and/or chemotherapy: 37 cases. They all had at least a 6-month follow-up without therapy and a stable or vanishing lesion.

**Table 3:** Distribution of the definitive diagnoses in the categories.

Diagnostic category		No. of cases
High-grade tumour (WHO III–IV) or metastasis		95 (46%)
Low-grade tumour (WHO I–II)		43 (21%)
Non-neoplastic		70 (33%)
	Reaction to irradiation and/or chemotherapy	37
	Inflammation or demyelinisation	14
	Focal epilepsy	4
	Genetic disorder	3
	Ischaemia	3
	Abscess	2
	Miscellaneous single lesions	7
**Total**		**208** (100%)

How often the diagnosis was correct, indeterminate, or incorrect when MRI was used alone and when MRS information was added is seen in Table 4. The number of indefinite diagnoses was lower and the number of incorrect diagnoses was higher when information from MRS was used, but that difference did not reach statistical significance (p = 0.055). MRI alone and supplemented with MRS provided a diagnosis belonging to the correct diagnostic category as often (62% vs. 64%, *p* > 0.2). However, the patients given a correct diagnosis were not always the same with these two methods. Both methods pointed to the same category in only 151/208 cases (73%). If both methods indicated the same category, the category was correct in 109/151 (72%).

**Table 4:** Outcome of the radiological diagnostics of the lesions. Comparison between the diagnoses from MRI only and when MRS information was added.

Radiological outcome	MRI N (%)	MRI + MRS N (%)	No. of cases with the same diagnostic outcome with both methods
Correct	130 (62%)	134 (64%)	109
Indeterminate	39 (19%)	23 (11%)	12
Incorrect	39 (19%)	51 (25%)	30
**Total**	**208 (100%)**	**208 (100%)**	**151 (73% of 208)**

No significant difference between the outcomes for MRI and MRI + MRS, p = 0.055

Distribution of the correct, indeterminate, and incorrect diagnoses in some subcategories can be seen in Table 5. The proportion of correct diagnoses, regardless of the method used, was lower in primary high-grade tumours (45–52%) than in the other subcategories (≥70%). The number of abscesses (*n* = 2) was too low to estimate diagnostic outcome. For the primary high-grade tumours (*n* = 89), the added information from MRS resulted in fewer indeterminate diagnoses but increased the proportion of incorrect diagnoses. In this group of tumours, MRI completed with MRS did not yield a correct category more often than MRI alone (52% vs. 45%, *p* > 0.2). Distribution of radiological diagnoses in cases with a confirmed diagnosis of primary high-grade CNS tumour is shown in Table 6. In only six patients, the lesion was a metastasis from an extracranial tumour. Five of them were classified as having high-grade tumours (Table 7), but one was incorrectly diagnosed with both methods. Two patients had an abscess. MRI gave the correct specific diagnosis in both, but MRS in only one. In the other case, abscess and glioma grade III were given as alternatives (Table 5). This patient had been treated with antibiotics prior to the radiological examinations.

**Table 5:** Outcome of the radiological diagnostics in some subcategories.

Definitive diagnosis	Radiological outcome	Modality	
		MRI	MRI + MRS
Primary CNS tumour WHO grade III–IV	Correct	40 (45%)^a^	46 (52%)^a^
	Indeterminate	23 (26%)	6 (7%)
	Incorrect	26 (29%)	37 (41%)
*Total*		*89 (100%)*	*89 (100%)*
Tumour WHO grade I–II	Correct	30 (70%)	30 (70%)
	Indeterminate	5 (12%)	7 (16%)
	Incorrect	8 (18%)	6 (14%)
*Total*		*43 (100%)*	*43 (100%)*
Reaction to irradiation/chemotherapy	Correct	29 (78%)	26 (70%)
	Indeterminate	8 (22%)	7 (19%)
	Incorrect	0	4 (11%)
*Total*		*37 (100%)*	*37 (100%)*
Inflammatory or demyelinating disease	Correct	11 (79%)	11 (79%)
	Indeterminate	2 (14%)	1 (7%)
	Incorrect	1 (7%)	2 (14%)
*Total*		*14 (100%)*	*14 (100%)*
Metastasis	Correct	5 (83%)	5 (83%)
	Indeterminate	0	0
	Incorrect	1 (17%)	1 (17%)
*Total*		*6 (100%)*	*6 (100%)*
Abscess	Correct	2	1
	Indeterminate	0	1
	Incorrect	0	0
*Total*		*2*	*2*

^a^ No statistically significant difference between MRI and MRI+MRS, p>0.2

**Table 6:** Distribution of radiological diagnoses to diagnostic categories in cases with a confirmed primary high-grade CNS tumour.

Radiological outcome	Radiological diagnosis	Modality	
		MRI	MRI+MRS
		N = 89	N = 89
**Correct category**	High-grade glioma	33	33
	High-grade glioma or metastasis	1	13
	Metastasis	6	0
	**Total**	**40**	**46**
**Indeterminate result**	High-grade or low-grade tumour	5	3
	High-grade tumour or non-neoplastic lesion	18	3
	**Total**	**23**	**6**
**Incorrect category**	Low-grade tumour	10	14
	Low-grade tumour or non-neoplastic lesion	6	7
	Non-neoplastic lesion	10	16
	**Total**	**26**	**37**

**Table 7:** Distribution of radiological diagnoses in cases with a confirmed metastatic disease.

Radiological outcome	Radiological diagnosis	Modality	
		MRI	MRI+MRS
Correct category	Metastasis	2	
	High-grade glioma or metastasis	1	3
	Glioblastoma	1	1
	High-grade tumour	1	1
Incorrect category	Abscess	1	
	Low-grade tumour		1
**Total**		6	6

Comparison of the radiological outcomes with the different spectroscopic methods are shown in Table 8. The use of the combined methods SVS and CSI did not improve radiological outcome compared to the cases where only one of the methods was used. However, the use of CSI gave additional information of the extensiveness and inhomogeneity of the lesions in certain cases.

**Table 8:** Radiological outcome with the different spectroscopic methods.

Radiological outcome	SVS	CSI	SVS + CSI
Correct	35	33	66
Indeterminate	4	6	13
Incorrect	15	10	26
**Total**	54	49	105

No statistically significant differences when the outcomes for SVS + CSI were compared to SVS or CSI only: correct vs. the others: p-value of 0.81 and 0.59, respectively and incorrect vs. the others: p-value of 0.68 and 0.55, respectively.

The additional information from MRS was considered to be very beneficial in seven cases (3%), beneficial in 24 (12%), inconsequential in 141 (68%), and misleading in 36 (17%). More detailed information is presented in Table 9. For selected indications, information from MRS was considered beneficial or very beneficial, e.g. in all four examinations performed to localise an epileptic focus.

**Table 9:** Clinical benefit of the added MRS compared to MRI only.

Value of added MRS	Type of additional information	Final diagnosis or diagnosis category	No. of cases N (%)
Very beneficial	Yielded a correct diagnosis category	Non-neoplastic	1
		Low-grade tumour	2
		High-grade tumour	1
	Revealed a lesion not found on MRI	Epileptic focus	3
		*Total*	*7 (3%)*
Beneficial	Excluded reaction to irradiation/chemotherapy	High-grade tumour	7
	Excluded tumour recurrence	Reaction to irradiation/chemotherapy	4
	Excluded incorrect tumour category	Low-grade tumour	2
		High-grade tumour	3
	Included correct diagnosis category among differential diagnoses	Non-neoplastic	1
		Low-grade tumour	1
		High-grade tumour	1
	Larger tumour extent than on MRI	Glioblastoma	2
	Excluded abscess from differential diagnosis	Glioblastoma	2
	Lateralization between bilateral lesions on MRI	Epileptic focus	1
		*Total*	*24 (12%)*
Inconsequential	No obvious additional information		141 (68%)
Misleading	Incorrect diagnosis category	High-grade tumour (including recurrent tumour)	9
	Included tumour in differential diagnosis	Reaction to irradiation/chemotherapy	7
	Less specific information on non-neoplastic lesions	Inflammation, metabolic disease and ischemia	5
	Excluded high-grade tumour from differential diagnoses	High-grade tumour	4
	Less specific information on tumour type	Oligodendroglioma	1
		Metastasis	2
		Glioblastoma	1
	Included incorrect tumour grade in differential diagnosis	Low-grade and high-grade tumour	3
	Excluded low-grade tumour from	Low-grade tumour	2
	differential diagnoses		
	Excluded reaction to irradiation from differential diagnoses	Reaction to irradiation	1
	Included tumour in differential diagnosis	Abscess	1
		*Total*	*36 (17%)*

## Discussion

In this study in a non-selected group of patients, the addition of MRS resulted in no improvement of categorization into non-neoplastic disease, low-grade tumour, or high-grade tumour compared to MRI only (64% vs. 62%). In the largest subgroup, primary CNS tumours grades III–IV, the difference between the methods was also non-significant (52% vs. 45%). If both methods indicated the same category, it was correct in 72% of cases. When the usefulness of a diagnostic method is evaluated, the proportion of correct diagnoses is not the only factor to consider because other clinically important information, such as the extent of a non-enhancing tumour/tumour component and locations of hot spots, can be obtained. At times, ruling out a specific diagnosis can be clinically valuable. In 15% of our cases, MRS was beneficial, providing more clinically important information than MRI.

Clinical MRS is often used when MRI has provided ambiguous results. This makes the accuracy low for MRI and may also affect the combination of MRI and MRS. The great majority of the earlier studies were performed in patients with tumours, often with selected groups of tumours. One-third of our lesions were non-neoplastic, in many cases without mass effect or contrast enhancement. Therefore it is not possible to make direct comparisons between our results and those in earlier studies. In the study of mass lesions by Möller-Hartmann et al. [2], 34/164 cases were non-neoplastic, and the proportion of correct diagnoses increased from 55 to 70% when MRS was added. However, 25 of their non-neoplastic lesions were abscesses, while there were only two in our series. MRS is an efficient tool to diagnose non-treated abscesses [11,19], but has been replaced by DWI in most clinical routines. In some studies, tumour grading using MRS has not been significantly better than MRI alone [6,7,9]. In some other studies, the diagnostic outcome has been better when MRS has been added, at least in some groups of tumours, but the statistical significances of the differences have not been reported [1,5,12]. In a recent study of 120 brain tumours in children [20], a correct tumour type was assigned statistically significantly more often with use of additional information from MRS than with that from MRI alone (87% vs. 71%). However, the criteria for a correct diagnosis were not the same in their and our study. They classified the diagnosis as correct if it was the only or most likely diagnosis. We classified the diagnosis as correct if it was the only diagnosis, but indeterminate if more than one alternative was given. Thus, they had a higher proportion of diagnoses called “correct” irrespective of the method used. We feel that from a clinical point of view, it is good to make a difference between the cases with one diagnosis which is correct and cases with many alternative diagnoses even if one of the diagnoses is correct.

The clinical impact of MRS in diagnostically difficult cases with indeterminate MRI findings has not been studied in large groups of patients. In case series consisting of 10–26 patients, MRS made significant contribution in 23–70% [5,21–23]. However, in one of those studies, histopathology did not support the MRS diagnosis in 4/16 cases in which preoperative MRS had had impact on decision-making [5]. All of these series have been very selective. In our more heterogeneous group of patients, clinically important additional information was obtained in only 15%. In some subgroups, the results were better, but the numbers of cases were very small. MRS provided e.g. more information in all four patients with refractory epilepsy and negative MRI and positron emission tomography (PET). Good results with MRS have been reported particularly in temporal lobe epilepsy [24,25]. Our experience with MRS in refractory epilepsy is limited because we routinely proceed with fluorodeoxyglucose and flumazenil PET if dedicated MRI is negative. In 141/208 (68%) of our cases, MRS did not give obvious additional information compared to MRI. Sometimes confirmation of the diagnosis with another method may have supported the clinician in decision making but it is difficult to assess in how many cases that really might have had clinical impact.

The largest category in this study was high-grade tumours (46%). The great majority of them were primary tumours and only six cases were metastatic. One explanation for the low number of metastases is that patients with more than one intracranial lesion were not examined with MRS if a primary tumour was diagnosed. MRI combined with MRS had low accuracy for the exact diagnosis of metastases, but the category (primary high-grade CNS tumour or metastasis) was correct in 5/6 cases. Results have varied when MRS has been used to differentiate between high-grade gliomas and metastases. Some authors have described patterns for this differentiation [26–29], whereas others have not been able to separate these groups successfully [30–33].

One common clinical problem is the differential diagnosis between a recurrent tumour and a reaction to tumour therapy, including radiation necrosis. This was the indication for MRS in one-third of our cases even though many such patients had to be excluded because of the difficulty in making a definitive diagnosis at a specific point of time. The situation is complicated even when samples have been taken for neuropathology because the lesion may be a mixture of neoplastic and reactive components.

In our study, we simulated the clinical setting at our department, i.e. MRS is performed in collaboration between a physicist and a neuroradiologist but the neuroradiologist is responsible for the diagnosis. At some centres, even the interpretation of MRS is only done by physicists. Neuroradiologists most likely utilise MRI information more than physicists when they interpret MRS. A combination of MRI and MRS has given better results than MRS alone [34]. The experience of the reader affects results both on MRI and MRS. We found a good interobserver agreement between assessments of the two experienced reviewers.

The MRS technique affects the results. In clinical circumstances, the choice of technique depends on the indication for the examination. The examination time and the patient´s state can be limiting factors. Our standard technique was a combination of SVS with a short TE and CSI with a semi-long TE and it was used in 50% of examinations. In that way, we could achieve good metabolite information, evaluate a large area and use two TEs. Significant differences between MRS with different TEs have not been found in all studies, but in some studies, the short TE has given better results, or the combination of two TEs has been recommended [26,28,31,35,36]. CSI shows heterogeneity of the tumours and can reveal hot spots for biopsy and neuropathological sampling. CSI is also more accurate in defining distinct tumour boundaries, as shown by histopathologic analyses in untreated patients with low- and high-grade gliomas [37]. In one-third to one-half of the patients with a suspected glioma, a tumour spectrum has been found outside the enhancement [38,39]. In the other half of our patients, only SVS, sometimes using two TEs or two locations, or CSI was successfully performed. We found no significant difference in diagnostic outcome when using SVS together with CSI compared to using only one spectroscopic method. The two-method technique was beneficial in cases when a good spectral quality was not reached with one method but the other method was successful. Metabolic ratios measured at SVS and CSI have shown a strong correlation in pontine gliomas even under treatment [40].

The number of patients included in our study is larger than in the studies referred to, but the numbers of patients with specific diagnoses are still small. That is why the statistical comparisons have been made only on a category level. The restriction to use only cases with well-confirmed diagnoses lowers the number of potential cases and can cause bias. In cases where the final diagnosis is based on clinical follow-up including MR examinations, the interpretation of those examinations may have influenced the definitive diagnosis. One factor with an influence is the interpretation of MRI and MRS together, but our intention was to make an evaluation using all available information, and not to test each technique as such. The placement of the voxel affects the results on MRS. The placement can be suboptimal if susceptibility effects or fat contamination is to be avoided. Heterogeneity of the lesion may affect both MRS and neuropathological diagnoses, as proven in gliomas [40,41]. If the area with pathological spectra were larger than the enhancement in patients with a verified neoplasm the interpretation was that MRS showed a larger extent than MRI. In our material, biopsies were not performed in those non-enhancing areas, but biopsy-verified tumour tissue outside enhancing areas and verified by CSI have been described in literature [37]. Therefore we interpreted that MRS provided beneficial additional information in these cases. We did not use a control voxel in an exactly symmetric location contralaterally to the lesion examined. In SVS, we mainly used a white matter voxel in the material of healthy volunteers. If CSI was performed in a region difficult to shim for susceptibility effects, e.g. close to the skull and in operated areas, the examination area was reduced and concentrated most on the suspected and abnormal area. Radiation therapy affects metabolite profiles. Radiotherapy is planned individually and the radiologist and physicist did not know how much, if any, radiation was applied in the certain regions outside the original neoplasm. We restricted postprocessing of CSI data to the range of 1.1–3.5 ppm. If the lactate peak (at 1.3 ppm chemical shift) is extremely wide the lactate quantification might be minimally affected but we do not believe that this has influenced diagnosis.

In conclusion, MRS did not add to the diagnostic value of MRI in general but yielded beneficial additional information in 15% of cases in clinical circumstances. To include MRS as a routine part of brain MR examinations does not seem to be indicated, but it can be useful in selected cases and may help in evaluation of disease extent or location of hot spots.

## Supporting information

S1 Table Principles of the MRS analysis. (DOCX)
