# Supplementary material for: The value of magnetic resonance spectroscopy as a supplement to MRI of the brain in a clinical setting
Source: PLoS One. 2018 Nov 15;13(11):e0207336. doi: 10.1371/journal.pone.0207336 (PMC6237369; doi:10.1371/journal.pone.0207336)
Supplement: S1 Table — (DOCX) [file pone.0207336.s001.docx]

**S1 Table. Principles of the MRS analysis**

| **MRS with a confirmed diagnosis, examinations with a low spectral quality excluded N=208** | | | | |
| --- | --- | --- | --- | --- |
| **SVS (TE 20-30 ms) N=54**  **Additional SVS (TE 135-141 ms) N=10** | | **SVS (TE 20-30 ms) + CSI (TE 135-144 ms) N=105**  All CSI voxels were analyzed | | **CSI (TE 135-144 ms) N=49**  All voxels were analyzed |
|  | | If the findings on SVS and CSI differed SVS findings were prioritized except with cases in which the region most pathological on CSI was outside the SVS. | | Extensiveness and heterogeneity of the pathological area were evaluated.  The most pathological (most malignant) spectrum was used for the final diagnosis. |
|  | | | | |
| **Guidelines for analysis of SVS**  **with a short TE** | | Examples and clarifications | Etiology | |
| Identification of main metabolites | |  |  | |
|  | Is something lacking? | No creatine peak | Creatine deficiency syndromes | |
|  | Existence of abnormal peaks | Succinate and acetate | Abscess (untreated) | |
|  | | Lactate (not abnormal in newborns)  Sign of anaerobic metabolism  To confirm that the peak in the lactate area is really lactate, additional CSI or SVS with a semilong TE should be available. If an inverted peak is not found the peaks at that area are lipids and amino acids. | Very high in necrosis.  Increased in malignant tumours (highest in Gr IV) and ischemic areas.  Can be increased in mitochondrial diseases | |
| Evaluation of main metabolites | |  |  | |
|  | NAA | Reduced if neuronal function or the number of the neurons are lowered | Slight decrease common. Strongly reduced in metastases and highly malignant tumours e.g. glioblastoma. May even be lacking in necrotic areas. NAA is reduced in irradiated brain tissue, slight reduction may be permanent. | |
|  | Choline | Reflects membrane turn-over | Increased in neoplasms (most in malignant tumours) but also in infections, inflammations etc. Cho is higher than normal in irradiated tissue. | |
|  | Creatine | The most stabile metabolite | Reduced in metastases and highly malignant tumours | |
|  | Myoinositol | Existent only in glia cells, mostly astrocytes | Increased in glial tumours, also in benign gliosis, inflammations etc. | |
|  | Lipids |  | Increased in tumours correlating with the grade of malignancy.  Very high in necrosis, abscesses | |
| **SVS and CSI with a semilong TE** | | Main differences to a short echo time spectra:  Myoinositol and lipids not seen  Lactate peak inverted |  | |
|  | |  |  | |
| Ratios between main metabolites | |  |  | |
| SVS with a short TE | | Ratios to Cr calculated for all metabolites  Ratios to Cho in selected cases (tumours) | Some border values:  High-grade vs. low-grade tumour:  High grade if Cho/NAA >1.6, Cho/Cr >1.56  Recurrent tumour vs. radiation injury:  Semilong TE: Cho/NAA and Cho/Cr >1.7  Short TE: Cho/Cr >1.4 | |
| CSI with a semilong TE | | Routinely calculated:  Ratios of Cho, NAA and lactate to Cr  Ratios of NAA, Cr and lactate to Cho |  |  |
|  | |  |  | |
| The diagnoses were not based on isolated findings as in one abnormal ratio; the spectrum was rather always handled as a combination of many metabolites and metabolite ratios, e.g. signs of a highly malignant tumour are reduced NAA, increased Cho, high lipids and high lactate. | | | | |
